# Involving older people in a multi-centre randomised trial of a complex intervention in pre-hospital emergency care: implementation of a collaborative model

**DOI:** 10.1186/s13063-015-0821-z

**Published:** 2015-07-10

**Authors:** Marina Koniotou, Bridie Angela Evans, Robin Chatters, Rachael Fothergill, Christopher Garnsworthy, Sarah Gaze, Mary Halter, Suzanne Mason, Julie Peconi, Alison Porter, A. Niroshan Siriwardena, Alun Toghill, Helen Snooks

**Affiliations:** Institute of Life Science, College of Medicine, Swansea University, Swansea, UK; School of Health and Related Research, University of Sheffield, Sheffield, UK; London Ambulance Service, London, UK; Community Library Service (Home Visits) Hackney Libraries, London, UK; Faculty of Health, Social Care and Education, Kingston University and St George’s, University of London, London, UK; Community and Health Research Unit, School of Health & Social Care, University of Lincoln, London, UK

**Keywords:** Consumer participation, Consumer involvement, Clinical trials, Service user involvement, Emergency medical care, Pre-hospital emergency care

## Abstract

**Background:**

Health services research is expected to involve service users as active partners in the research process, but few examples report how this has been achieved in practice in trials. We implemented a model to involve service users in a multi-centre randomised controlled trial in pre-hospital emergency care. We used the generic Standard Operating Procedure (SOP) from our Clinical Trials Unit (CTU) as the basis for creating a model to fit the context and population of the SAFER 2 trial.

**Methods:**

In our model, we planned to involve service users at all stages in the trial through decision-making forums at 3 levels: 1) strategic; 2) site (e.g. Wales; London; East Midlands); 3) local. We linked with charities and community groups to recruit people with experience of our study population. We collected notes of meetings alongside other documentary evidence such as attendance records and study documentation to track how we implemented our model.

**Results:**

We involved service users at strategic, site and local level. We also added additional strategic level forums (Task and Finish Groups and Writing Days) where we included service users. Service user involvement varied in frequency and type across meetings, research stages and locations but stabilised and increased as the trial progressed.

**Conclusion:**

Involving service users in the SAFER 2 trial showed how it is feasible and achievable for patients, carers and potential patients sharing the demographic characteristics of our study population to collaborate in a multi-centre trial at the level which suited their health, location, skills and expertise. A standard model of involvement can be tailored by adopting a flexible approach to take account of the context and complexities of a multi-site trial.

**Trial registration:**

Current Controlled Trials ISRCTN60481756. Registered: 13 March 2009

## Background

There is increased expectation that people who use health and care services should be involved in research including trials, but little evidence that this is happening in practice. Though service user involvement in health services research is growing, the majority of studies in which service users are involved are qualitative, rather than trials [1–3].

UK government policy requires involvement by service users in all research undertaken through the National Health Service (NHS) [4–8] and most national and international research funding programmes expect service users to be involved in the development and conduct of studies which are submitted to them [9–11]. The network of UK Clinical Trials Units encourages service users to work with researchers in designing and undertaking trials (see http://www.ukcrc-ctu.org.uk/?page=Patients).

Service user involvement in research has been defined as research carried out ‘with’ or ‘by’ members of the public rather than ‘to’, ‘about’ or ‘for’ them [12]. Involving service users in research is encouraged to improve relevance, quality and accountability of research [12–16].

Guidelines recommend ways to enhance involvement to support effective input from service users and improve opportunities for positive impacts on research [17–19]. Guidance has also been published to support their involvement in trials [20]. Researchers are advised to adopt a flexible approach to facilitate and enhance involvement, by considering *‘different approaches within the same trial or group of trials, involving different people in different ways at different stages’* [21]. Involvement can take place at all research stages through consultation, collaboration or by service users leading the research [12].

A systematic review published in 2011 identified only 9 studies reporting service user involvement in the design and conduct of clinical trials. Challenges reported in these trials included: identifying service users who were representative of trial participants, particularly people who were older and frail; facilitating public understanding of trial methodology; and ensuring a participatory framework which enabled active involvement within trial resources [22]. A bibliometric literature review identified the most common health topic research areas to involve service users as: mental health; health of black, minority, ethnic and indigenous groups; children and parenting. In contrast, the health of older people was found to be among the research topics least likely to involve service users [2]. Additionally, no reviews have identified studies reporting the involvement of service users in pre-hospital and emergency care research [2, 23, 24].

Factors which enhance the quality and impact of service user involvement in research include: clarifying the role of service users; acknowledging their skills, knowledge and experience; providing support, information and resources to enable involvement; involving people at an appropriate level; describing and acknowledging service user involvement in study reports [25, 26]. It is challenging to involve service users who are harder to reach because of illness, age, cultural background or geography [27]. Researchers can endeavour to ensure that involvement is accessible to all by targeted recruitment and addressing barriers caused by lack of mobility, income, information and skills [28].

The SAFER 2 trial (Support and Assessment for Fall Emergency Referrals) evaluated the clinical and cost-effectiveness of referral to community-based falls services by paramedics for older people who had fallen but did not require care at the emergency department (ED) [29]. The trial, funded by the National Institute of Health Research Health Technology Assessment (NIHR HTA) programme in response to an unsustainable rise in emergency ambulance demand for non-life-threatening emergencies, was a multi-centre pragmatic cluster-randomised controlled trial (C-RCT) led from Swansea University, with 25 participating ambulance stations across 3 study sites in Wales, London and East Midlands.

As an NIHR-funded trial, SAFER 2 was required to ensure that members of the public were actively involved throughout the research process. SAFER 2 was supported by the Swansea Clinical Trials Unit (CTU) which developed, as one of its suite of Standard Operating Procedures (SOPs), a SOP for involving service users in trials [20]. This SOP outlined roles and processes for public involvement and recommended involving service users in all trial decision-making forums, including specific recommendations for service user representation (a minimum of two people) on structures such as the Trial Development Group (TDG) and Trial Management Group (TMG). The SOP also made recommendations for support and remuneration to service user members and was the basis for facilitating service user involvement in the SAFER 2 trial. This paper describes how we implemented a collaborative model to involve older people in the SAFER 2 multi-centre trial of a complex intervention in pre-hospital emergency care.

## Methods

We took the Swansea CTU SOP for service user involvement in trials [20] as our starting point. The SOP recommends levels of service user involvement in a trial at strategic management level (such as TMG) and at local management level. It also suggests researchers identify opportunities for service user input through ad hoc task-focused meetings. We considered that this approach required modification to suit the multi-centre structure of our trial, which included site level (Wales, London, East Midlands) and local levels of implementation (delivering the intervention on the ground to patients). At study outset, the study team agreed a plan. A researcher led its implementation and oversaw site researchers who had responsibility for service user involvement in their own areas. Advice and guidance was provided by the CTU Involvement Manager about developing and implementing the model, recruiting and supporting service users.

### Proposed structures of involvement

We developed a model for involvement that took into account the multiple layers of a multi-site trial and allowed active involvement in overall or specific aspects of the trial. We included service users at the three levels: 1) strategic 2) site and 3) local. We defined strategic level as relating to overall management of the multi-centred trial. We defined site level as relating to one of the three regions which constituted a study site: Wales; London; East Midlands. We defined local level as relating to implementation of the intervention to the patient in the community.

At Levels 1 and 2, we invited service users to be involved in the structures which we set up to manage the project:

- At Level 1, we aimed to recruit service user members to join the strategic level study meetings – TMG, Trial Steering Committee (TSC) and Data Monitoring and Ethics Committee (DMEC). All trial co-applicants and staff were invited to the TMG, where members took decisions and oversaw study management and implementation. The TSC and DMEC were independent oversight groups acting on behalf of the Project Sponsor and Project Funder to ensure the project was conducted to a rigorous standard. We included two service users on each of these groups [6].
- At Level 2, we recruited service users to join a Site Management Team (SMT) at each site to oversee implementation and delivery of the trial by bringing together local service users and study partners involved with delivering the intervention and collecting data at each site.

At Level 3, we created a Service User Reference Group (SURG) in each site. This had not been part of the SOP and was a new structure in our model, established in order to liaise with service users outside the formal management structures of the trial and provide a forum for service user discussion and contribution to aspects of the study.

In line with the SOP and good practice [12], we aimed to involve 2 service users in each of these structures at Level 1 and Level 2 and up to 10 individuals in each SURG (Level 3). Service users could attend meetings at different levels, if they wished, but TSC and DMEC members needed to remain independent of the trial management and implementation. We intended to involve service users in research processes at all stages of the trial: developing and planning research; data collection; analysis; and dissemination.

### Recruiting and engaging service users

The complex intervention being evaluated by SAFER 2 – to prevent recurrent falls – was targeted at older people, who were likely to be frail or had a history of falling and whose use of services was unplanned and urgent. In order to identify service users (patients, carers and people at risk of falling) with these characteristics, we linked with local organisations, charities and community groups (for example, Stop Falls Network charity; Hackney Housebound and Mobile Library Service; Age Concern). We asked them to distribute information about the research opportunity and encouraged organisers to identify potential individuals in order to reach people with relevant experiences, appropriate ages and different backgrounds. We provided training and support to enable people to be involved and set aside funding within the study budget to host meetings, pay honorariums and reimburse costs incurred through involvement [30].

### Data collection and analysis

We collected notes of meetings and other documentary evidence including attendance records, drafts of study papers and accounts of research tasks, to describe numbers of service users involved, level and research processes they were involved in, to illustrate how we implemented our model to involve service users in the SAFER 2 trial. We compared implementation with planned involvement to consider issues, lessons learned and to identify areas where service users contributed to the trial. We involved service users from the SAFER 2 trial in writing this paper by discussing drafts, inviting comments and including feedback, to ensure this account accurately reflected all experiences. Results are reported for the trial period 1 April 2009 to 23 May 2014, presenting an account of how involvement began, how it evolved over time and how experience varied from intention. Results are presented for: structures of involvement; recruitment and participation by service users; and processes of involvement undertaken. This format reflects the first two elements of the three factors – context, mechanism and outcome – whose links should be explored when assessing public involvement in research [23].

## Results

### Structures of involvement

**Fig. 1:**
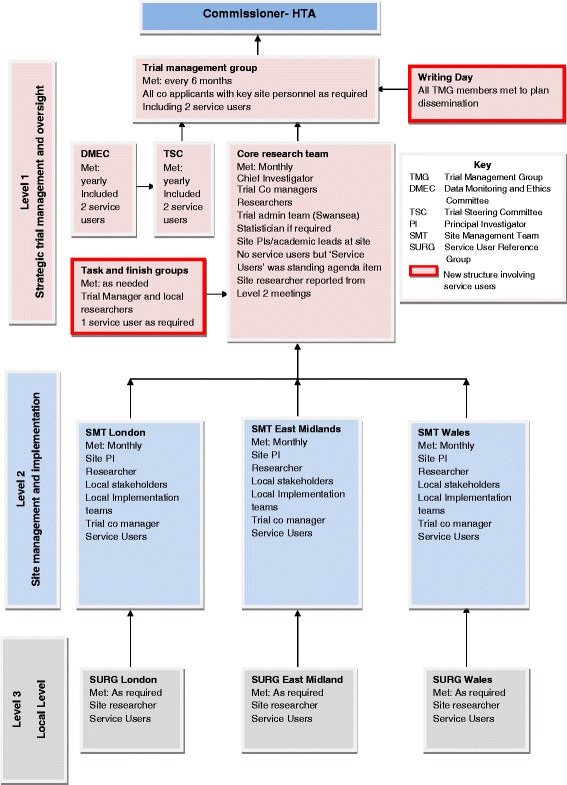
Overall management flowchart of Support and Assessment for Fall Emergency Referrals (SAFER 2) showing structures where service users were involved

The management structure of the SAFER 2 trial is shown in Fig. 1. Structures which were not originally planned are outlined in bold in order to distinguish planned from actual arrangements. Service user involvement at and within each level is shown. In addition to the involvement structures planned at study outset, we created Task and Finish (T&F) Groups at strategic level and convened Writing Days throughout the study to plan dissemination, to which all TMG members including service users were invited.

### Recruitment and participation by service users

At the strategic level (Level 1), we aimed to involve 2 service users in each meeting of the TMG, DMEC and TSC. However, levels of participation were not consistent over the 5 years, with variation in attendance and changes in the individuals involved. Attempts to identify a third, reserve service user to attend when a regular member was unable, were only partially successful because service users reported they found it difficult to sustain the levels of knowledge and sense of engagement when patterns of involvement were irregular. Service users took part in three of five T&F Groups to: discuss ways of improving response rates to the patient questionnaires; plan and undertake qualitative analysis; hold a one-off monitoring meeting with the HTA funder. We also convened a group to oversee the procedural process of gaining Research and Development permissions; and a technical group to develop the research database. Because of the specialist and technical nature of these two groups, we decided not to involve service users. We held three Writing Days, attended by none, two and one service users, respectively. The first concentrated on developing a publication plan; the second on publications for academic journals and the final report; the third exclusively on the final report.

At the site level (Level 2), service user recruitment was challenging and attendance was inconsistent. Site researchers, who were responsible for recruiting service users to SMT meetings, found this task competed with other research duties (including support for the SURGs). It was also hampered by researchers’ levels of confidence and a turn-over of site researchers during the trial and periods between appointments when there was no site researcher. No service users were involved in site management meetings in East Midlands. One service user joined site management meetings in Wales in the last year of the trial. Attendance at London site management meetings was variable, with one early attendee finding the meeting format overwhelming and attendance onerous because of other caring responsibilities. This coincided with the departure and replacement of the site researcher.

At the local level (Level 3), involvement varied according to the success of different approaches to recruiting members to the SURGs. In Wales, the site researcher reconvened, for one meeting, a group which met for a previous study on a similar topic area [29]. In East Midlands, members of a local service user group, the Sheffield Emergency Care Forum (see www.secf.org.uk) met once as part of SAFER 2. In London, the site researcher used skills developed in a previous role and contacts with local charities and service providers (Stop Falls Network charity and Hackney Library Service) to recruit six service users with direct and relevant experience. The group was initiated in the third year of the study and met five times until the study end. SURG members’ involvement included helping to design data collection tools, developing patient information and piloting patient interviews. SURG meetings were held at universities (Wales and East Midlands) and community venues (London).

Participation in the 3 levels of involvement is summarised in Table 1.

**Table 1:** Participation in involvement structures in SAFER 2

Type of meeting	Number of meetings held	Number (%) of meetings with service users present	Comments
Level 1: Strategic involvement			
Trial Management Group (TMG)	10	8 (80%)	Two service users were invited to each meeting
Trial Steering Committee (TSC)	4 (1 per annum)	3 (80%)	Initially, one invitee per meeting. Increased to two after second meeting with no service user present
Data Monitoring and Ethics Committee (DMEC)	6 (1 per annum)	6 (100%)	Also increased to two per meeting to keep consistent with TSC
Task and Finish (T&F) Group	5	3 (60%)	Two T&F Groups considered very technical and not appropriate for service user involvement
Writing Day	3	2 (66%)	Service user members of TMG were invited to the writing days
Level 2: Site level - Site Management Teams			
Wales	18	4 (22%)	One service user attended meetings in later stages of the study
East Midlands	8	0	No service users recruited
London	9	3 (33%)	Initial and later service user attendance
Level 3: Local level - Service User Reference Groups (SURGs)			
Wales SURG	1	1	Eight members met once. Recruited from panel co-ordinated by Age Concern for SAFER 1 study
East Midlands SURG	1	1	Four members met once. Recruited through Sheffield Emergency Care Forum – service user group linked to Sheffield University
London SURG	7	7	Six members met 7 times in years 3, 4, 5. Recruited through Falls Prevention Service, Housing Association, mobile library service for housebound people

Study researchers reported that contacts with service users involved in SAFER 2 increased as the research team developed ongoing relationships with individual service users. Researchers identified further opportunities to enable service users to contribute to the study through the T&F Groups and Writing Days. The team tried to make meetings as accessible as possible. We paid honoraria and expenses, we chose accessible venues and we always provided refreshments. Teleconferencing and Skype were available for anyone unable to travel. Because our aim was to ensure service users could contribute to different research processes through the 3 levels (see Table 1), we were flexible and opportunist in order to capture and consider their views. For example, when poor health prevented a service user member of the TMG from attending meetings, a researcher arranged meetings at his home to gain his views on the data collection tools under discussion. Links between involvement levels and reporting mechanisms are shown in Fig. 1.

### Research processes in which service users were involved

We involved service users in research processes at all stages of the research journey: planning and managing research; data collection; data analysis; dissemination. Strategic and study-wide decisions such as agreeing research methods, undertaking analysis and dissemination took place at TMG or strategic T&F meetings. In-depth discussions on recruitment, data collection and data management at SMT meetings, and SURG meetings especially, enabled service users to contribute to the detail of research, such as by role-playing the interview respondent or suggesting ways to improve questionnaire response rates by advising on wording on the patient questionnaire and using coloured paper to ensure it remained obvious and memorable to patients when mixed within a pile of other papers. In the qualitative analysis T&F Group, the service user received guidance and support in order to carry out the same tasks as other team members, reviewing data and highlighting themes and key points for coding data. In addition to identifying themes that were similar to those identified by other team members, he also noted additional themes that the research team had not identified. For example, his patient and carer perspective prompted him to identify the cost of mobility aids as a theme emerging from interview data. All service users were encouraged to be involved in writing this paper, through discussions at SURG meetings and commenting on circulated drafts, in writing or discussion. Research processes which service users were involved in are shown in Table 2. In some cases, the same issues were discussed at more than one level.

**Table 2:** Support and Assessment for Fall Emergency Referrals (SAFER 2) research processes involving service users

Research stage	Research process	Involvement structure
Planning and managing research	Ensuring delivery of safe, high-quality research against agreed study objectives and timescales	Level 1
		TMG, DMEC, TSC
	Attending the monitoring meeting by study funder	Level 1
		T&F one-off meeting
	Reviewing site study progress against timescales	Level 2
		SMT
	Developing research database	Level 1
Data collection	Developing patient questionnaire and interview schedule	Level 1
		TMG
		Level 2
		SMT
	Reviewing and refining draft patient interview schedule	Level 1
		TMG
		Level 3
		SURG
	Reviewing and refining draft patient questionnaires	Level 3
		SURG
	Identifying ways to improve the completion rate of the patient consent form	Level 1
		T&F Group
		Level 3
		SURG
	Piloting patient interviews	Level 3
		SURG
	Reviewing and amending site data management challenges	Level 2
		SMT
	Developing patient thank you letters	Level 3
		SURG
Analysis	Reading patient transcripts to identify themes for analysis	Level 1
		T&F analysis Group
	Coding data from patient transcripts	Level 1
		T&F analysis Group
	Inputting into interpretation and presentation of findings from patient interviews	Level 1
		T&F analysis Group
Dissemination	Commenting on drafts of final report	Level 1
		TMG
	Contributing to papers, conference presentations and final report	Level 1
		Writing Day
	Contributing to this paper	All service users from Levels 1, 2, 3

*DMEC* Data Monitoring and Ethics Committee, *TMG* Trial Management Group, *TSC* Trial Steering Committee, *SURG* Service User Reference Group, *T&F* Task and Finish, *SMT* Site Management Team

## Discussion

### Summary of results

The SAFER 2 trial involved service users in decision-making forums across all levels of research management and implementation at all study sites. Service users were involved at strategic and site levels through management and oversight meetings; at local level through SURGs; and additionally through other task-related meetings. These routes allowed service users, who reflected our vulnerable patient group, to contribute insight to research tasks which researchers would not have gained otherwise. However, involvement was uneven with variation in number and frequency of service user involvement across all meetings, study levels and study locations. Some of this was due to health and availability of service users and we adapted ways to account for this while maintaining our aim to achieve cross-study involvement. Researcher confidence, skills, competing research tasks and availability also limited where and how much we were able to involve service users. That we partially achieved the plan for involvement across all three study sites raises the question of how feasible it actually was to ensure service user collaboration at every stage and forum of the trial. Within our plan, adapted from our CTU’s SOP for service user involvement, we identified and implemented new opportunities for service user involvement by creating the SURG. Our approach also evolved in response to the study context and attitudes of researchers, so that we created service user places in T&F Groups and at Writing Days.

### Strengths and limitations

We have given a descriptive account of how we developed and implemented the SAFER 2 involvement model. Researchers and service users have co-authored this paper. We were not able to collect and report data about the quality of involvement or the experiences of service users, researchers and other partners involved in the trial. These issues were not considered in the planning stages of the trial and, while funding was available to actively involve service users, we did not have a budget to assess processes and experiences concerning this aspect of the study. Thus, we have described, but not evaluated, our involvement model. There remains an opportunity to assess its ability to achieve service user collaboration in a trial and its impact on the research.

### Implications for practice

Our involvement model provided a framework for service user involvement in a multi-centre trial which can be used in other studies. It highlighted facilitators and barriers affecting the involvement process which we have summarised in Table 3. The model incorporated two types of involvement, which we call ‘inreach’ and ‘outreach’ types of involvement. In ‘inreach’ approaches, service users were invited to become partners in the management structures for the trial at strategic or site level. ‘Outreach’ approaches occurred when members of the research team went out to service users in the SURGs to involve them at local level. Links were maintained between the three levels of involvement by the three site-based researchers and through reporting mechanisms. The ‘inreach’ opportunities appealed to some service users, who chose to enter into the world of the research teams; for others, the ‘outreach’ opportunities offered a chance to take part in less formal settings and generally among their peers. The type of setting particularly suited our hard to reach population of older, frail, emergency ambulance service users, who we accessed through our links with local champions. Having the SURGs allowed us to meet these service users more on their terms. Our findings echo advice from INVOLVE [21] and from Tritter and McCallum [31] who reported: *‘effective user involvement must be founded on connections to a multiplicity of individuals and groups and the integration of one-off and more continuous involvement’.* Rhodes et al. [32] found that hard to reach users of diabetes services reported feeling more able to honestly advise researchers about evaluation when they met as a separate group.

**Table 3:** Facilitators and barriers to involvement through the Support and Assessment for Fall Emergency Referrals (SAFER 2) model

Facilitators
• Model for multi-level involvement providing structure for flexible approach which adapted to circumstances during the study
• ‘Inreach’ and ‘outreach’ involvement opportunities provided opportunities to suit different service users’ interest and experience
• Research team support enabled implementation of model
• Service user interest and commitment supported involvement across the study
• Site-based researchers facilitated and supported service user involvement
• Financial resources were available to cover involvement costs
Barriers
• Service users’ health and existing commitments limited their availability
• Technical, scientific and timetable requirements in undertaking and delivering research tempered service users’ motivation
• Continuity of involvement due to time between meetings and fluctuating research pace across research stages hindered ability to sustain involvement
• Changes in site researchers and time to recruit qualified replacements reduced resources to support service user involvement leading to involvement gaps at some levels and sites
• Research confidence, skills and competing research duties

Involving service users in trials has not been widely reported despite recommendations to involve service users in all types of studies [12, 21]. In SAFER 2, we aimed to involve service users through collaboration by supporting and enabling their active involvement in trial decision-making forums. Active involvement at all stages of a trial is the ideal but not always practicable and feasible for people experiencing poor health, unfamiliar with academic terminology or the detail of research processes. It has also been reported to cause stress, illness and just be unachievable for some individuals [27, 33, 34]. The more regular engagement in the London SURG, compared to the other two areas, suggests that tailored support and facilitation skills in the research team may be important in achieving regular attendance and input to a trial [24, 27, 28]. Service users are a naturally diverse population and optimal approaches to involvement will vary accordingly [35]. Our experience also confirms that service user involvement in trials is strengthened over time as relationships, skills and knowledge are developed and sustained.

### Implications for research

Boote et al. [22] found that lack of a participatory framework limited involvement in trials. The SAFER 2 model is a framework, within which innovation (such as the SURG) can occur, be documented and assessed. It provides a structured approach to developing knowledge about extending and enhancing involvement in different contexts.

Service users want to influence practice and improve services for future patients [34, 36, 37]. In our experience, the extended periods between developing a research idea, preparing and submitting a proposal and gaining funding can frustrate and temper motivation. Trials can take several years to complete, further testing sustained involvement. Meanwhile, our experience suggests that the process of involvement benefits from continuity over the period of a trial. The time and input demanded of such a commitment can be onerous for patients and their carers. Future studies should prospectively record and evaluate the involvement processes in a trial to identify the most effective ways of sustaining collaboration and how this affects the research. Resourcing and planning for evaluation should occur alongside study development. Work to develop and report measures of effective involvement is advancing [25, 38–41] and should be considered when planning, evaluating and reporting involvement in trials.

## Conclusion

In the SAFER 2 trial, we have shown how it is feasible and achievable for patients, carers and potential patients to collaborate in a multi-centre trial of a complex intervention. A standard model of involvement can be tailored to suit any particular trial and a flexible approach can assist in incorporating the complexities of a multi-site study. Our approach allowed variations and adjustments to be made to the model across study sites and study levels. Tiers of involvement provided opportunities for people to be involved at strategic, site or local level where it best suited their health, location, skills and expertise. However, we found attendance at meetings was difficult for some service users, thus showing a need for flexibility, commitment and skills in the study team to sustain inclusion. We offer our involvement experience as a model for other trials to base their work on and from which to extend and evaluate collaboration with service users in trials.

## Abbreviations

C-RCT: cluster-randomised controlled trial
CTU: Clinical Trials Unit
DMEC: Data Monitoring and Ethics Committee
ED: emergency department
NHS: National Health Service
NIHR HTA: National Institute for Health Research Health Technology Assessment
SAFER: Support and Assessment for Fall Emergency Referrals
SMT: Site Management Team
SOP: Standard Operating Procedure
SURG: Service User Reference Group
T&F: Task and Finish
TDG: Trial Development Group
TMG: Trial Management Group
TSC: Trial Steering Committee
